# Antimicrobial activity of medicinal plants used for urinary tract infections in pastoralist community in Ethiopia

**DOI:** 10.1186/s12906-021-03249-7

**Published:** 2021-02-23

**Authors:** Eshetu Gadisa, Elazar Tadesse

**Affiliations:** 1 Department of Medical Laboratory Science, Menelik Medical and Health Science College, Kotebe Metropolitan University, P.O. Box 3268, Addis Ababa, Ethiopia; 2 Department of Nutrition Science, MMHSC, KMU, Addis Ababa, Ethiopia

**Keywords:** *Cirsium englerianum*, *Cucumis pustulatus*, *Discopodium penninervium*, Multidrug resistance

## Abstract

**Background:**

Medicinal plants have wide medicament application used to prevent and management of many ailments. These plants are used for primary health care in pastoralist communities who are deprived of modern medical care. They possess extensive therapeutics bioactive coupled with varied chemical structures. However, scientific validation of efficacy and safety of plants used to treat the urinary tract infections haven’t been fully exploited. The aim of this study was to evaluate antimicrobial activity and screening phytochemicals of medicinal plants used to treat urinary tract infections.

**Methods:**

In-vitro experimental study was carried out to evaluate the antimicrobial effect and screening phytochemical of *Rumex abyssinicus, Cucumis pustulatus, Discopodium penninervium, Lippia adoensis, Euphorbia depauperata,* and *Cirsium englerianum.* Against drug resistance microbes. 80% methanol was used for extraction of the plant parts. The susceptibility tests were investigated using disc diffusion and broth micro-dilution methods.

**Results:**

The majority of tested extracts showed antimicrobial activity on two or more drug-resistant bacteria with MIC value (1.0–128.0 μg/ml) and 9–27 mm inhibition zone in diameter. Extracts obtained from *C.englerianum* and *E. depauperate* showed more potent antibacterial activity on MRSA and *Enterococcus faecalis* with IZ 25 and 27 mm respectively*. E. coli* and *K. pneumoniae* were inhibited by those extracts with IZ ranging 9–25 mm and 11–27 mm respectively. *E.faecalis* and *K. pneumoniae* were more susceptible bacteria to the respective extracts. *R. abyssinicus* showed promising antifungal effect with had 21 mm IZ and MIC range 16-32 μg/ml on *C.albicans*. Alkaloids, flavonoids, phenolic and terpenoid were common phytochemical characterized in majority of screened plants.

**Conclusion:**

Tested extracts exhibited significant antibacterial and antifungal activity. Hence, further structural elucidation of bioactive that inhibited the growth of microbes aforementioned plants may be used as precursors for the synthesis of new antibiotics in the future.

## Background

Over the past several decades urinary tract infection is one of the most common infections. It is mostly caused by gram-negative bacteria namely *E.coli P. aeruginosa* and *P.neumoniae* [1]. There are also urinary tract infection (UTI) caused from *S. aureus* and *E. faecalis* and *C. albicans* in immunocompromised patients and pregnant women [2]. On the top of this, emerging and re-emerging of MRSA, extended-spectrum β-lactamases producing organisms (ESBL), vancomycin-resistant enterococci (VRE), and the carbapenem-resistant organisms (CRE/CRP) is a global public health challenge and imposed socioeconomic crisis worldwide [3, 4]. WHO report showed overuse and misuse of available antibiotics and lack of discovery of the new antimicrobials drug by the pharmaceutical industry make the crisis more sever and life threaten condition UTI infected patients [5].

On the other hand, many resistance genes/ gene products are speed in bacteria population through efflux, hyper-mutability and plasmid addiction. Such factors attributed for compromised all or majority of the drugs belonging to a given therapeutic [6]. As a result, urinary tract infections caused with resistant organisms have higher morbidity and mortality, are costlier to treat, result in longer hospital stays and place a greater burden on health systems than infections caused by susceptible organisms. Many studies shown that in United States, at least 2 million people acquire serious infections with bacteria that are resistant to one or more of the antibiotics used for the treatment of infections [7]. The total economic cost of antibiotic resistance was estimated as high as $20 billion in direct healthcare and $35 billion in lost productivity per year. If there were no successful efforts to cure them or no new drugs to combat them, the number of deaths per year would be ten million and the cost would increase up to $100 trillion by 2050 [8]. So, searching for innovative antibiotics from natural products should be ultimately an important segment of modern medicine to overcome the various socioeconomic and health impacts caused by multidrug resistant microbes [9].

Searching of medicinal plants used as alternatives or complementary treatment of emerging and re-emerging of multidrug resistance bacteria is undergoing a revitalization because it is available, accessible, affordable and acceptable to the local population [10]. According to WHO report, medicinal plants can potentially meet communities needs and improve access to safe, quality and culturally sensitive primary health care. Which in turn, they can make a significant contribution to essential health services in the prevention and management of communicable diseases caused by drug resistance bacteria [11]. Many studies have shown that each medicinal plant has many phytochemical compounds namely coumarins, flavonoids, phenolic, alkaloids, terpenoids, tannins, essential oils, lectin, polypeptides, and polyacetylenes. The presence of these bioactive compounds can show bactericidal or bacteriostatic effects on multidrug resistance pathogen bacteria and also as precursor for develop antibiotics for treating infectious agents, mainly from urinary tract infections causing bacteria [12–14].

Likewise, antibacterial potent of crude extract obtained from *Bidens sulphurea, Bidens pilosa,* and *Tanacetum vulgare* demonstrated antibacterial action on *S. aureus, E. faecalis, E.coli,* and *P. aeruginosa* isolated from UTI patients and standard bacterial strains ranged from 7.81-125 mg/l and the MBC values ranged from 7.81–500 mg/l [15]. *Calotropis gigantean* has shown no antibacterial and antifungal activities for n-hexane fractions against pathogenic microbes; however, its ethyl-acetate fractions how potential inhibitory effects on all tested bacteria and fungi except *T.rubrum* [16].

Another study conducted on the organic extracts of *Phragmanthera capitat* had comparative antimicrobial activities against ten pathogen bacteria with a MIC of 1.25–5 mg/ml and MBC of 2.5–10 mg/ml and fungi species with a MIC of less than 0.3125 to 1.25 mg/ml [17]. Likewise, the aqueous extracts of *Carum copticum,* and *Albizia adianthifolia* showed significant antibacterial effect against multidrug resistance gram negative human pathogen bacteria such as *E.coli, Pseudomonas, K. pneumonia,* Salmonella and Proteus species [18, 19]. Several aqueous and hydro-alcoholic extracts obtained from different plants were screened for their antibacterial effects against human pathogenic bacteria and demonstrated remarkable antibacterial effects on multidrug resistant bacteria including MRSA and ESBL producing bacteria [20–24]. Moreover, a significant number of plants exhibited a broad antibiotic spectrum against multidrug resistant bacteria, which could help to control the problem of the global threat imposed by infections due to multidrug resistant bacteria [25, 26].

It has been pointed out that chemical synthesis and the search for natural products from living organisms (marine and higher plants) are the two major sources of new bioactive compounds. However, less than 1% of plants were isolated and characterized for their secondary metabolites and used for pharmacological utilization [27, 28]. Traditional medicinal plants are the most valuable sources of new bioactive chemical entities due to their biodiversity coupled with the chemical diversity found within each species [29–33]. In line with this, *C.englerianum, E.depauperata, L adoensis, D.penninervium, C.pustulatus* and *R.abyssinicus* are TMPs used by communities for treatment of different infectious agents. Yet, they haven’t been thoroughly examined for their antimicrobial potential and phytochemical entities as therapeutic agents for multidrug resistant bacteria and human pathogen fungi.

## Methods

### Study area and design

#### Study area

This study was conducted in the Seweyna Woreda, Bale Zone. This Woreda is located 437 km away from Robe (Zonal Town) in the Northern east direction and 750 km from the capital city, Addis Ababa. An elevation extends from 400 to 1850 m above sea level and is located at coordinates latitude 7° 19′ 60.00“ N and longitude 41° 19’ 60.00” *E. major* rivers include the Mekenisa, Dare, Manduba, and Kurkura. According to the national land survey showed arable or cultivable (24.4%), pasture (46.3%), forest or heavy vegetation, and forest (24.1%) of the land. This district is the remotest area with no infrastructure (transport, hospital, and power supplies). Since there was only one health center in the Woreda, the residents depend on traditional knowledge of medicinal plants to treat human ailments such as skin diseases, diabetes, UTI, hepatitis, sexual transmitted infection, cancer, hypertension, impotence, and contraceptive.

#### Study design and period

In vitro, an experimental study was carried out to evaluate the antimicrobial effect and screening phytochemical of *C.englerianum, E.depauperata, L adoensis, D.penninervium, C.pustulatus* and *R.abyssinicus* against clinically isolated multidrug-resistant bacteria and their reference strains, and pathogen fungi at KMU, MMHSC, Core Laboratory from January to April, 2020.

### Medicinal plants selection criteria

Nowadays, communities are deprived of modern medical care and depend on the traditional medicinal plants to treat many human and animals’ ailments such as skin diseases, diabetes, urinary tract infection, hepatitis, sexually transmitted diseases, cancer, hypertension, sexual impotence, and contraceptive. Of which, *C.englerianum, E.depauperata, L adoensis, D.penninervium, C.pustulatus* and *R.abyssinicus* are commonly used to treat bacterial infection such as eczema, gonorrhea, syphilis, pneumonia, scabies, skin infection, and superficial mycosis.

### Plant collection and extraction

The leaf, bark, or root parts of *C.englerianum, E.depauperata, L adoensis, D.penninervium, C.pustulatus* and *R.abyssinicus* was collected and screened for their antibacterial and fungi and phytochemical. Authentication of this plant sample was carried out and deposited at the National Herbarium, Department of Biology, Faculty of Natural Science and computational Science, Addis Ababa University. The fresh parts of the plant were collected and washed thoroughly under running tap water and dried in dark shaded areas. The air-dried plant materials (4 kg each) was powdered and extracted at room temperature with 80% methanol and distilled water. Triplicate of 200 g portions of the dried powdered plant was soaked separately in 1000 ml of distilled water, or methanol for 3 days with frequent agitation and the resulting liquid was filtered using Whatman No.1 filter paper (Whatman Ltd., England). Extraction was repeated seven times and the filtrates of all portions were combined in one vessel. The methanol was removed by evaporation using Rota vapor at 40 °C. After the removal of the organic solvent, the aqueous residue was placed in lyophilizer until non-polar solvents removed and dried the extracts. The resulting dried mass packed into a glass vial and stored in a desiccator over silica gel until use [13, 32].

### Sterility test of extracts

Each extract of methanol and aqueous was tested for the growth of bacteria. This was carried out by inoculating 0.5 ml of each of them on sterile Mueller Hinton Agar and incubated at 37 °C for 18–24 h. The plates were observed for growth. No growth in the extracts after incubation indicated that they were sterile and evaluated for antibacterial activity as described by CLSI guideline [32].

### Culture media and multidrug bacteria

#### Culture media

Nutrient agar, TSY broth, MacConkey, Muller Hinton agar, Muller Hinton Broth, blood agar, mannitol salt agar, chocolate agar and biochemical reagents for bacteria and sabouraud dextrose agar for fungi were obtained from the Departments of Medical Laboratory Science, Bacteriology Unit.

#### Test organisms

The reference bacterial species of *E. coli* (ATCC25922), *K. pneumoniae* (ATCC700603), *Enterococcus faecalis* (ATCC 29212) and *Staphylococcus aureus* (ATCC 25923) and their MDR strains, and fungi i.e. *Candida albicans* reference (ATCC 10535 and those obtained from sample) were used for this study [32].

#### Modern antibiotics

Currently available antibiotics; ciprofloxacin(5 μg), gentamycin (10 μg), cephalotaxon (30 μg), cefotaxime (5 μg), ceftazidime (10 μg), cefoxitin (30 μg), ceftriaxone (30 μg), amikacin (30 μg), cefuroxime (5 μg), ceftriaxone (30 μg), cloxacillin (5 μg), and augmentin (30 μg) were used to test for bacteria and ketoconazole (20 μg) for *C. albicans* as stated in the CLSI guideline [32].

### Screening for multidrug resistant bacteria

Multidrug resistant gram negative and gram-positive bacteria were isolated from different samples (urine, throat swab and blood). All bacterial cultures were first grown on 5% blood agar plates at 37°C for 18-24hrs prior to inoculation onto the MHA. Few colonies (3-5) of similar morphology of the respective bacteria were transferred with a sterile inoculating loop to a liquid medium until adequate growth of turbidity with McFarland in 0.5. Then the bacterial suspension was streaked on MHA plates using a sterile swab in such a way as to ensure thorough coverage of the plates and a uniform thick lawn of growth following incubation. The susceptibilities of clinical isolates were tested by using the MHA contains a range of antimicrobial agents. Dilutions of overnight broth cultures were inoculated onto antibiotic containing plates to yield final inoculums of approximately 10^6^ CFU per spot for *Enterobacteriaceae*. Selected multidrug resistant *K. pneumoniae* and *E. coli* were screened for their resistant for more than two different classes of antibiotics following the disk diffusion method as CLSI guideline [13, 31, 32].

### Determination of MIC and MBC values

After preliminary screening of plants for their antimicrobial activity, those revealed potent antimicrobial effects were further tested to determine MIC and MBC against multidrug resistant gram negative, and gram-positive species. It was determined by MHB broth micro-dilution method. Each 96-well microtiter plate was liquated with 50 μl of MHB; 10th well (sterility control) was added with 100 μl of MHB. And the 9th well (growth control) was added with MHB with 5% DMSO. 50 μl of each extract initially dissolved in 5% DMSO was added into the first well. A serial 2-fold dilution was performed by transferring suspension to the subsequent wells up till the 8th well; this procedure was performed by CLSI guideline and modifying Wiegand protocol [25]. 0.5 McFarland broth inoculum was diluted in the ratio of 1:100 and added into 1st- 8th well in achieving the final inoculum size at 5 x 10^5^CFU/ml [28].

Bacterial cell viability and MIC values were determined by observing the turbidity. The lowest concentration of the extract with clear suspension was considered as the MIC values. The lowest concentration of the extract in the post-incubation suspensions which did not harbor any bacterial growth upon spotting on MHA after overnight incubation at 37 °C were considered as the MBC values. The test was performed in triplicates alongside antibiotics ciprofloxacin (5 μg) as a positive control at 0.1 mg/ml [27, 28]. MIC values were determined by observing the turbidity. The lowest concentration of the extract with clear suspension was considered as the MIC values. The lowest concentration of the extract in the post-incubation suspensions which did not harbor any bacterial growth upon spotting on MHA after overnight incubation at 37 °C were considered as the MBC values. The same method as for bacterial tests was adopted for fungi. However, instead of nutrient agar, sabouraud dextrose agar was used. The inoculated medium was incubated at 37 °C for *C. albicans.* The test was performed in triplicates alongside antibiotics ciprofloxacin (5 μg) as a positive control at a concentration of 0.1 mg/ml for bacteria; whereas, ketoconazole was used as a positive control at a concentration of 0.3 mg/ml for fungi [13, 31, 32].

### Phytochemical screening

The presence of the major phytochemical constituents was identified as alkaloids, flavonoids, saponins, phenolic, tannins, terpenoid, and cardiac glycosides in 80% methanol extracts of each plant using modified standard procedures as mentioned our previous work [13, 33].

### Statistical analysis

The triplicate data were reading values of inhibition zones in diameter and concentration values (MIC & MBC) analyzed using SPSS, version 21 according to CLSI. Each experiment values were expressed as mean ± SD. Statistical significance was determined by student’s T-test. Values with *p* < 0.05 were considered significance.

## Results

### Phytochemical screening of medicinal plants

This study revealed that air dried powder form of each medicinal plants has varies yield in 80% methanol extraction. The highest yield was observed for *C. englerianum* (38%) which used to treat gonorrhea, tonsillitis, syphilis, wound amebiasis, malaria, and diarrhea by pastoralist community. However, the lowest yield was observed for *E. depauperata* (22%) which was mainly used to treat skin rash, ringworm, bloody diarrhea, gastritis, and constipation (Table 1). On the other hand, the qualitative phytochemical investigation showed that these tested medicinal plants have different phytochemicals namely; saponins, tannins, alkaloids, terpenoids, anthraquinones, phenolics, cardiac glycosides and flavonoids (Table 2). Thus, naturally occurring bioactive attribute significant bactericidal action on (*E. coli, K. pneumoniae, S. aureus,* and *E. faecalis)* and fungicidal (*C. albicans)* properties.

**Table 1:** Ethno-botanical data and percentage yields of medicinal plants tested on pathogenic microbial

Scientific name	Family	Local name	Part used	% yield (mean ± SD)	Locally used to treat
*Cirsium englerianum*	Asteraceae	Adaddoo	Leaf	38.1 ± 1.0	Gonorrhea, UTI, tonsillitis, syphilis, wound amebiasis, malaria, diarrhea
*Euphorbia depauperata*	Euphorbiaceae	Gurii	Bark	22.3 ± 0.9	Skin rash, ringworm, bloody diarrhea, UTI, gastritis, constipation
*Lippia adoensis*	Verbenaceae	Urgoo	Leaf	27.3 ± 1.7	Stomach ache, kidney disease, diarrhea, wound and cough
*Discopodium penninervium*	Solanaceae	Maraaro	Leaf	33.9 ± 0.4	Eczema, wound, scabies, urine retention
*Cucumis pustulatus*	Cucurbitaceae	Haadhatu	Root	29.1 ± 2.8	Cough, TB and chest pain, cold disease, Pneumonia
*Rumex abyssinicus*	Polygonaceae	Dhangagoo	Root	24.0 ± 0.3	Gonorrhea, skin diseases, diarrhea, abscesses, ringworm, pain-relieving, diuretic effect, hypertension, anti-cancer, malaria, wound healing

**Table 2:** Preliminary phytochemical screening of some traditional medicinal plants, KMU, 2020

Scientific name (parts)	Solvent used	Major phytochemicals in crude extract of medicinal plants							
		Saponin	Tannins	Alkaloids	Terpenoids	Anthraquinone	Flavonoids	Phenolic	Cardiac glycoside
*C. englerianum* (fruit)	Methanol	+	++	+++	+	+	+++	+++	++
	Aqueous	–	++	++	+	–	+	+++	++
*E. depauperata* (Bark)	Methanol	+++	–	++	+	+	+++	+	+
	Aqueous	++	–	+	–	++	++	++	–
*L. adoensis* (Leaf)	Methanol	++	+	+++	+	+++	++	+	–
	Aqueous	–	+	+	–	+	++	+	+
*D.penninervium* (Leaf)	Methanol	+++	+	+++	+	++	+++	–	++
	Aqueous	+	+++	++	+	++	+	–	+
*C. pustulatus* (Root)	Methanol	+	++	–	–	++	+	++	+
	Aqueous	+	_	–	+	+	+	+	++
*R. abyssinicus* (Root)	Methanol	++	+	+	++	+++	+	++	–
	Aqueous	+++	+	++	+	++	–	++	+

*+++ = Appreciable amount, ++ = Moderate amount, + = Trace amount, − = Not detected*

### Antibacterial effect of extracts on multidrug resistant bacteria

Almost all extracts showed antibacterial activity against two or more of the multidrug resistant and reference strains of human pathogenic bacteria. The extract of *E. depauperata, R. abyssinicus* and *L. adoensis* showed promising antibacterial activity as they inhibited the growth of *K. pneumoniae, S. aureus* and *E. faecalis* with zone of inhibition ranging 21-27 mm at their 30 μl/disc. The methanolic extract of *C. englerianum, L. adoensis* and *E. depauperata* showed inhibition zone of 25 mm, 22 mm and 23 mm in diameter against *MRSA* respectively. Of gram-positive bacteria, *E. faecalis* was highly susceptible for methanolic extract of *C. englerianum.* This extract inhibited the growth of both drug resistant and susceptible strains with 27 mm inhibition zone. Moreover, the effectiveness of tested extract had MIC value of gram-positive bacteria ranging 1.0–64.0 μg/ml. In this regard, *C. englerianum* showed bactericidal in the low dose on *E. faecalis* with MIC and MBC values 1.0 μg/ml and 2.0 μg/ml respectively. Likewise, it inhibited the growth of *S. aureus* with 24 mm inhibition zone, MIC and MBC values were 16 μg/ml and 32.0 μg/ml respectively (Table 4).

The methanolic extract of *E. depauperata* had strong antibacterial activity on MSSA and MRSA with inhibition zone of 23 mm (Table 3). This plant extract exhibited remarkable inhibition of the growth of MRSA with MIC and MBC values ranging from 4.0–8.0 μg/ml (Table 4).

**Table 3:** Inhibition zone in diameter of pathogenic microbes on agar disc diffusion, KMU, 2020

Scientific name	Concentration		Inhibition zone in diameter (mm)									
			Gram positive bacteria				Gram negative bacteria				Fungi	
			*S. aureus*		*E. faecalis*		*E.coli*		*K. pneumoniae*		*C. albicans*	
	Mg/ml	μl/disc	ATCC	MRSA	ATCC	MDR	ATCC	MDR	ATCC	MDR	ATCC	Sample
*C. englerianum*	100	15	16 ± .0.1	15 ± 0.4	18 ± 0.9	17 ± 1.0	9 ± 0.3	13 ± 1.4	16 ± 2.0	11 ± 0.1	14 ± 1.1	–
		30	24 ± 3.0	25 ± 0.8**	27 ± 1.3	27 ± 1.5^#^	20 ± 0.7	21 ± 0.2	26 ± 0.8	26 ± 0.9^¥^	21 ± 0.7	9 ± 0.3
*E. depauperata*	100	15	15 ± .0.6	14 ± 0.7	16 ± 2.0	17 ± 0.3	–	–	17 ± 0.1	18 ± 0.7	–	10 ± 0.9
		30	23 ± 2.4	23 ± 1.0*	26 ± 0.9	26 ± 0.5^#^	13 ± 0.1	12 ± 0.4	25 ± 1.2	25 ± 0.7^¥^	22 ± 1.1	23 ± 0.7
*L. adoensis*	100	15	16 ± .1.2	12 ± 0.1	11 ± 1.1	14 ± 0.1	10 ± 0.7	14 ± 0.2	20 ± 0.6	20 ± 0.3	–	9 ± 0.3
		30	22 ± 2.4	21 ± 1.8	21 ± 0.5	21 ± 1.5	21 ± 2.1	21 ± 0.3	27 ± 0.1	27 ± 0.7^¥^	11 ± 0.9	20 ± 0.1
*D.penninervi m*	100	15	16 ± .0.3	14 ± 0.1	16 ± 0.4	15 ± 0.7	20 ± 0.7	19 ± 1.0	18 ± 0.3	16 ± 0.2	13 ± 0.3	9 ± 1.6
		30	21 ± 0.5	23 ± 2.0*	21 ± 0.1	20 ± 1.0	25 ± 0.8	23 ± 0.1	23 ± 0.7	21 ± 0.4	20 ± 0.9	19 ± 0.4
*R. abyssinicus*	100	15	14 ± 0.9	9 ± 0.8	15 ± 1.1	11 ± 1.0	–	–	11 ± 1.0	9 ± 0.7	10 ± 0.2	14 ± 0.4
		30	22 ± 0.6	20 ± 0.4	25 ± 0.4	19 ± 1.1	10 ± 0.8	12 ± 0.1	20 ± 0.1	19 ± 0.6	21 ± 0.5	21 ± 0.1
*C. pustulatus*	100	15	12 ± 1.0	12 ± 0.2	12 ± 0.8	16 ± 1.2	15 ± 0.2	10 ± 0.8	10 ± 0.9	11 ± 0.8	12 ± 0.8	10 ± 0.1
		30	21 ± 0.8	21 ± 0.8	23 ± 0.5	22 ± 0.8^#^	23 ± 0.4	18 ± 0.9	21 ± 1.0	21 ± 0.4	18 ± 0.1	18 ± 0.8
Modern drug			22 ± 2.0^Ce^	12 ± 1.9^Ce^	22 ± 3.0^C^	13 ± 1.2^C^	26 ± 2.1^C^	15 ± 2.3^C^	24 ± 1.2^C^	14 ± 1.9^C^	22 ± 0.3^F^	21 ± 1.3^F^

*Mean ± SD, NT* Not tested*, − = No inhibition zone, C* Ciprofloxacin*, Ce* Cefoxitin*, F* Ketoconazole, MDR < 16 mm, ATCC > 21 mm *P < 0.05, **P < 0.01 compared to cefoxitin treated MRSA. ^*#*^*P < 0.05 compared to modern drug treated E. faecalis,* ^*¥*^ *P < 0.05 compared to modern drug treated K. pneumoniae*

**Table 4:** MIC and MBC/MFC values of methanolic extract against pathogenic microbes, KMU, 2020

Scientific name	MIC/MBCMFC	Concentration of extracts in μg/ml on pathogen bacteria and fungi									
		Gram positive bacteria				Gram negative bacteria				Fungi	
		*S. aureus*		*E. faecalis*		*E.coli*		*K. pneumoniae*		*C. albicans*	
		ATCC	MRSA	ATCC	MDR	ATCC	MDR	ATCC	MDR	ATCC	Sample
*C.englerianum*	MIC	16.0	16.0	1.0	1.0	64.0	64.0	2.0	2.0	128.0	64.0
	MBC/MFC	32.0	32.0	2.0	2.0	128.0	128.0	2.0	2.0	128.0	128.0
*E. depauperata*	MIC	4.0	4.0	4.0	4.0	128.0	128.0	16.0	16.0	NT	64.0
	MBC/MFC	4.0	8.0	8.0	8.0	128.0	128.0	16.0	16.0	NT	128.0
*L. adoensis*	MIC	64.0	32.0	16.0	16.0	64.0	64.0	16.0	16.0	384.0	512.0
	MBC/MFC	128.0	128.0	16.0	16.0	64.0	64.0	32.0	32.0	NT	NT
*D. penninervium*	MIC	8.0	8.0	8.0	16.0	16.0	16.0	16.0	16.0	128.0	128.0
	MBC/MFC	16.0	16.0	8.0	8.0	16.0	16.0	32.0	32.0	128.0	128.0
*R. abyssinicus*	MIC	16.0	16.0	64.0	64.0	128.0	128.0	16.0	16.0	32.0	16.0
	MBC/MFC	32.0	16.0	128.0	128.0	64.0	64.0	32.0	16.0	64.0	32.0
*C. pustulatus*	MIC	64.0	64.0	4.0	4.0	32.0	32.0	8.0	8.0	128.0	256.0
	MBC/MFC	64.0	64.0	8.0	8.0	64.0	64.0	16.0	16.0	128.0	NT
Modern drug		0.1^Ce^	++	0.1^C^	++	0.1^C^	++	0.1^C^	++	0.3^F^	0.3^F^

*C = 5 μg ciprofloxacin, Ce* Cefoxitin*, F = 20 μg ketoconazole, ++ = Growth has been seen, NT* Not tested *MDR = < 16 mm, ATCC > 21 mm IZ*

This study revealed that *E. depauperata, C. englerianum* and *D. penninervium* inhibited the growth of *E. coli* and *K.pneumoniae* that attributed for 85% urinary tract infections. In line of this, those medicinal plants inhibited the growth of multidrug resistant *K. pneumoniae* ranging 11-27 mm in diameter. Of which, *L.adoensis* and *D. penninervium* are demonstrated promising treatment for multidrug resistant *K.pneumoniae* with 27 mm and 26 mm inhibition zone respectively (Table 3). On the other hand, *E. depauperata, C. englerianum* and *D. penninervium* demonstrated on the multidrug resistant *E. coli* with 12 mm, 21 mm and 23 mm inhibition zones in diameter respectively (Table 3). The second most susceptible bacteria were clinical isolates of multidrug resistant and reference strain of *K. pneumoniae* with MIC and MBC value ranging from 2.0–16.0 μg/ml and 2.0–32.0 μg/ml respectively (Table 4).

Overall, those medicinal plants have bioactive that able to bactericidal property and used as alternative and/or complementary treatment for urinary tract infection. This is illustrated by minimum bactericidal concentration as parallel tested with modern antibiotics. As a result, thoroughly exploited traditional medicinal plants is the way to overcome health consequence of drug resistance bacteria caused human and animals’ ailments in general and urinary tract infections in particular.

### Antifungal activity of medicinal plants

This study revealed that methanolic extracts obtained from *C. englerianum, L. adoensis, D. penninervium* and *R. abyssinicus* demonstrating remarkable anti-fungal effect against *C. albicans* (Table 3). Of which, *R. abyssinicus* showed promising antifungal effect as compared to others. It had 21 mm inhibition zone in diameter and MIC values ranging from 16 to 32 μg/ml. In addition, extracts of *D. penninervium* and *C. englerianum* showed inhibitory activity against fungi with MIC/MFC values ranging16-512 μg/ml, whereas those of *L. adoensis* was found to be less potent for *C. albicans* with MIC ranging 512 μg/ml.

## Discussion

Nowadays, majority of the cases from urinary tract infections caused by multidrug resistance microbes constitutes an important therapeutic challenge. They are widely spread and public health concern at global scale worldwide. Resistance mechanism developed by MDR microbes are transfer from one strain to another bacteria through efflux, hyper-mutability and plasmid addiction. As a result, urinary tract infections caused with MRSA, extended-spectrum β-lactamases producing organisms (ESBL), vancomycin-resistant enterococci (VRE), and the carbapenem-resistant organisms (CRE/CRP) have higher morbidity and mortality. They are costlier to treat, result in longer hospital stays and place a greater burden on health systems than infections caused by susceptible organisms. These multidrug resistant microbes have compromised all or majority of currently accessible and affordable antibiotics widely used in the developing countries. In such case, searching for antibiotic from natural resources in general and medicinal plants in particular is timely concern. Those plants have wide application in the treatment of urinary tract infections caused by multidrug resistant bacteria and fungi. This can be demonstrated in that almost all tested medicinal plants had shown promising antimicrobial activity against the selected human pathogenic microbes. This finding substantiates from previous studies that therapeutic agents derived from plants can be used as an important alternative for the treatment of infectious disease [9–12].

This study revealed that all species of the plants used here had antibacterial activity and also indicated that *E. faecalis* was the most susceptible species of all tested human pathogenic bacteria. Moreover, most of the extracts have shown remarkable inhibitory effect on MRSA. Likewise, multidrug resistant and its reference strains of *K. pnemoniae* were found to be the most sensitive gram-negative bacteria against the tested plant extracts. Implausibly, both multidrug resistant and reference strains of *E. coli* were the most unresponsive strain of all tested bacteria species. These findings agreed with several reports [13, 19–21] which demonstrated that gram positive (*S. aureus* and *E. faecalis*) bacteria to be more sensitive than gram negative (*E. coli* and *K. pneumonia*) bacteria for the plants’ phytochemical components being studied. Moreover, many studies have shown that medicinal plants contain different bioactive ingredients that inhibited the growth of human pathogenic bacteria [22]. On the other hand, researchers demonstrated that *E. coli* has developed multidrug resistance to many currently available and affordable antibiotics [3, 23]. Thus, genetic makeup to *E. coli* to produce multidrug resistance was favorable to impede the antibiotics through reduced permeability and efflux pump [6, 23, 26]. As a result, searching for antibacterial activity of the secondary metabolites from medicinal plants is a timely concern [12–14]. In this regard, medicinal plants can be used to overcome socioeconomic and health impact caused by multidrug resistant bacteria, including MRSA and multidrug resistant gram-negative bacteria such as *E. coli* and *K. pneumoniae* [12–14].

Regarding the human pathogen fungi, the presence of bioactive compounds in traditional medicinal plants could inhibit the growth of *C. albicans*. A study done by Shumaia Parvin shown that C*. gigantean* had inhibitory potential on all tested human pathogenic bacteria and fungi except *T. rubrum* [16]*.* Another study conducted on extracts of *Carum copticum and Albizia adianthifolia* had exhibited good antimicrobial activities against *E. coli, Proteus species, P. aeruginosa, P. neumoniae* and *Salmonellae species* [18, 19].

Overall, this study showed that among the tested human pathogenic microbes, fungi were found to be more unaffected by many of the tested extracts than bacteria. These findings demonstrated that *C. albicans* was less susceptible than those tested pathogen bacteria*.* The presence of different bioactive constituents can inhibit the growth of not only multidrug resistant bacteria but also inhibit growth of fungi. This difference in effect on the growth of *C. albicans* indicate the presence of antifungal constituents in the crude extracts of each plant. This agrees with previous findings on the antifungal activity of medicinal plants containing different secondary metabolites that inhibited pathogen fungi [16, 17, 27].

We can affirm that our preliminary findings suggest most endemic medicinal plants contain flavonoid, anthraquinones, alkaloids, tannin, phenolic and saponin (Table 2). The presence of these bioactive ingredients can inhibit the growth of not only reference strains but also multidrug resistant microbes. In this regard, many studies demonstrated that medicinal plants contain coumarins, flavonoids, phenolic, alkaloids, terpenoids, tannins and polyacetylenes which can be bactericidal, bacteriostatic or produce fungicidal effect on human pathogenic microbes [11, 13, 29]. Other researchers proposed that this inhibitory activity of secondary metabolites may be due to sequential inhibition of biochemical pathways, inhibition of protein synthesis and disintegration of the outer membrane of the microbes [20, 24]. In line with this, many researchers argued that medicinal plants contain diversified secondary metabolites and this makes them a possible alternative for treating infections caused by microbes including multidrug resistant microbes [13, 14, 17, 28]. Therefore, a lot has to be done to explore the potential of such medicinal plants as a possible source of antimicrobials that can be used against multidrug resistant bacteria and fungi.

## Conclusions

Those of tested medicinal plants were found to have more antibacterial effect than antifungal activity. They showed promising antimicrobial effect on multidrug resistant bacteria. Hence, identification and isolation of secondary metabolites from those medicinal plants will help pharmaceutical companies to develop modulator or precursor for the synthesis of new novel antibiotics used to treat diseases caused by pathogens including multidrug resistant bacteria.

## Data Availability

All data and materials of this work are available from the corresponding author on request.

## Abbreviations

ATCC: American type culture collection used as reference strains
CLSI: Clinical laboratory standard institute
ESBL: Extended spectrum beta lactamase
MBC: Minimum bactericidal concentration
MDR: Multi drug resistant
MHA: Muller hinton agar
MHB: Muller hinton broth
MRSA: Methicillin resistant *Staphylococcus aureus*
MSSA: Methicillin susceptible *Staphylococcus aureus*
MBC: Minimal bactericidal concentration
MIC: Minimal inhibitory concentration
TMP: Traditional medicinal plants

## References

[1] Fair RJ, Tor Y (2014). Antibiotics and bacterial resistance in the 21st century. Perspect Medicin Chem.

[2] Lafon T, Hernandez Padilla AC, Baisse A (2019). Community-acquired *S aureus* bacteriuria: A warning microbiological marker for infective endocarditis. BMC Infect Dis.

[3] Behzadi P, Behzadi E, Yazdanbod H, Aghapour R, Akbari Cheshmeh M, Salehian OD (2010). Urinary tract infections associated with *C. albicans*. Maedica.

[4] Heintz BH, Halilovic J, Christensen CL (2010). Vancomycin-resistant enterococcal urinary tract infections. Pharmacotherapy.

[5] Ludden C, Cormican M, Vellinga A, Johnson JR, Austin B, Morris D (2015). Colonization with ESBL-producing and carbapenemase-producing Enterobacteriaceae, vancomycin-resistant enterococci, and MRSA in a long-term care facility over one year. BMC Infect Dis.

[6] Shriram V, Khare T, Bhagwat R, Shukla R, Kumar V (2018). Inhibiting bacterial drug efflux pumps via phyto-therapeutics tocombat threatening antimicrobial resistance. Front Microbiol.

[7] Frost I, Van Boeckel TP, Pires J, Craig J, Laxminarayan R. Global geographic trends in antimicrobial resistance: the role of international travel. *J Travel Med*. 2019;26(8):taz036. 10.1093/jtm/taz036 31115466

[8] Prestinaci F, Pezzotti P, Pantosti A (2015). Antimicrobial resistance: a global multifaceted phenomenon. Pathog Glob Health.

[9] Dadgostar P (2019). Antimicrobial resistance: implications and costs. Infect Drug Resist.

[10] Brown E, Wright G (2016). Antibacterial drug discovery in the resistance era. Nature..

[11] WHO (2019). Global report on traditional and complementary medicine 2019.

[12] Rossiter SE, Fletcher MH, Wuest WM (2017). Natural products as platforms to overcome antibiotic resistance. Chem Rev.

[13] Gadisa E, Weldearegay G, Desta K (2019). Combined antibacterial effect of essential oils from three most commonly used Ethiopian traditional medicinal plants on multidrug resistant bacteria. BMC Complement Altern Med.

[14] Khameneh B, Iranshahy M, Soheili V (2019). Review on plant antimicrobials: a mechanistic viewpoint. Antimicrob Resist Infect Control.

[15] Liu HY, Lin HC, Lin YC, Yu SH, Wu WH, Lee YJ (2011). Antimicrobial susceptibilities of urinary extended-spectrum beta-lactamase-producing *E. coli* and *K. pneumoniae* to fosfomycin and nitrofurantoin in a teaching hospital in Taiwan. J Microbiol Immunol Infect.

[16] Shumaia P, Abdul K, Aktar C, Ekramul H (2014). Antibacterial, antifungal and insecticidal activities of the n-hexane and ethyl-acetate fractions of methanolic extract of the leaves of *Calotropis gigantea*. JPP.

[17] Ohikhena FU, Wintola OA, Afolayan AJ (2017). Evaluation of the antibacterial and antifungal properties of *Phragmanthera capitata* (Sprengel) Balle (Loranthaceae), a mistletoe growing on rubber tree, using the dilution techniques. Sci World J.

[18] Maheshwari M, Safar Althubiani A, Hasan Abulreesh H, Abul Qais F, Shavez Khan M, Ahmad I (2019). Bioactive extracts of *Carum copticum* enhances efficacy of ciprofloxacin against MDR enteric bacteria. Saudi J Biol Sci.

[19] Tchinda CF, Sonfack G, Simo IK (2019). Antibacterial and antibiotic-modifying activities of fractions and compounds from *Albizia adianthifolia* against MDR gram-negative enteric bacteria. BMC Complement Altern Med.

[20] Tayel AA, Shaban SM, Moussa SH, Elguindy NM, Diab AM, Mazrou KE, El-Sabbagh SM (2018). Bioactivity and application of plant seeds’ extracts to fight resistant strains of *S. aureus*. Ann Agric Sci.

[21] Marathe NP, Rasane MH, Kumar H, Patwardhan AA, Shouche YS, Diwanay SS (2013). In vitro antibacterial activity of Tabernaemontana alternifolia stem bark aqueous extracts against clinical isolates of methicillin resistant *S aureus*. Ann Clin Microbiol Antimicrob.

[22] Saeidi S, Amini Boroujeni N, Ahmadi H, Hassanshahian M (2015). Antibacterial activity of some plant extracts against extended- spectrum beta-lactamase producing *E. coli* isolates. Jundishapur J Microbiol.

[23] Terlizzi ME, Gribaudo G, Maffei ME (2017). Uropathogenic *E.coli* infections: Virulence factors, bladder responses, antibiotic, and non-antibiotic antimicrobial strategies. Front Microbiol.

[24] Chuah EL, Zakaria ZA, Suhaili Z, Abu Bakar S, MNM D (2014). Antimicrobial activities of plant extracts against MSSA and methicillin-Resistant S. aureus. Int J Microbiol Res.

[25] Gonzalez-Alamilla EN, Gonzalez-Cortazar M, Valladares B,. et al. Chemical constituents os Salix babylonica and their antibacterial activity against gram positive and gram negative animal bacteria. Molecules. 2019;24(16):2992. 10.3390/molecules24162992 PMC6721091 31426583

[26] Aris P, Boroumand MA, Rahbar M, Douraghi M (2018). The activity of fosfomycin against extended-spectrum beta-lactamase-producing isolates of Enterobacteriaceae recovered from urinary tract infections: a single-center study over a period of 12 years. Microb Drug Resist.

[27] Salam AM, Quave CL (2018). Opportunities for plant natural products in infection control. Curr Opin Microbiol.

[28] Varaprased B, Pacheco G, Alcantara C, Abreu C, Correa M (2012). A search for antimicrobial agents. Relationship between chemical structure and activity of triterpene against gram positive and gram-negative bacteria.

[29] Atanasov AG, Waltenberger B, Pferschy-Wenzig EM (2015). Discovery and resupply of pharmacologically active plant-derived natural products: a review. Biotechnol Adv.

[30] Katiyar C, Gupta A, Kanjilal S, Katiyar S (2012). Drug discovery from plant sources: an integrated approach. Ayu.

[31] Wiegand I, Hilpert K, Hancock R (2008). Agar and broth dilution method to determine the MIC of antimicrobial substances. Nat Protoc.

[32] CLSI (2011). Performance for antimicrobial susceptibility testing: twenty-first informational supplement-M 100-S21.

[33] Abdissa D, Geleta G, Bacha K, Abdissa N (2017). Phytochemical investigation of *Aloe pulcherrima* roots and evaluation for its antibacterial and antiplasmodial activities. PLoS One.

